# A Quantitative Method for 3D Scan Quality Assessment Under Different Surface Conditions for Reverse Engineering of Shipyard Components

**DOI:** 10.3390/s26051581

**Published:** 2026-03-03

**Authors:** Fabrizio Freni, Simone Panfiglio, Elnaeem Abdalla, Antonio Cannuli, Guido Di Bella, Roberto Montanini

**Affiliations:** 1 Department of Engineering, University of Messina, C.da di Dio, 98166 Messina, Italy; simone.panfiglio@itae.cnr.it (S.P.); elnaeem.abdalla@studenti.unime.it (E.A.); antonio.cannuli@unime.it (A.C.); guido.dibella@unime.it (G.D.B.); roberto.montanini@unime.it (R.M.); 2 NAVTEC Distretto Tecnologico Trasporti Navali, Via Comunale S. Lucia 40, 98125 Messina, Italy

**Keywords:** reverse engineering, measurement comparison, 3D scan, scan quality index, surface conditions

## Abstract

Shipyards are transitioning toward Industry 4.0 more slowly than other industrial sectors, and this inertia often limits the adoption of reliable digital workflows for reverse engineering. Within the wider research aimed at supporting the digital transition of shipbuilding operations, this study presents a dedicated methodology for evaluating 3D scan quality by combining three complementary indicators describing geometric completeness, agreement with a reference model, and measurement accuracy and variability. A purpose-designed test sample representative of shipbuilding geometrical challenges was manufactured in metal by CNC methods and in PLA through additive manufacturing. Two scanning systems, a field-oriented portable device and a metrology-oriented fixed system, were evaluated under raw surface conditions and with tracking enhancement strategies (optical markers and scanning spray). Results show that reflective surfaces represent a critical scenario, where tracking enhancement is essential to obtain continuous reconstruction and reliable dimensional correspondence. Conversely, with low-reflectivity surfaces, high-quality reconstructions can also be achieved with portable systems, with tracking enhancements mainly improving uniformity and repeatability. Overall, the proposed workflow provides a quantitative basis to support scanner selection, which involves a compromise between portability and achievable metrological performance, for shipyards reverse engineering applications.

## 1. Introduction

Shipyard environments are increasingly pressured to reconcile productivity, cost control and sustainability objectives while operating under constraints that are structurally different from those of highly automated serial manufacturing. The push toward “Shipyard 4.0” is not merely a technology trend, but reflects a concrete need to reduce rework, shorten downtime, and improve dimensional quality control across the lifecycle of vessels and ship systems, especially in contexts where documentation may be incomplete, outdated or unavailable after years of modifications and repairs. Within this perspective, digital transformation frameworks increasingly highlight the centrality of dimensional quality management as a prerequisite for data-driven planning and execution, and as a foundational layer for digital twin adoption in shipbuilding [1].

A recurring barrier in practice is the geometry gap between the physical asset and the information available to engineers and technicians. In shipbuilding and ship repair, this gap manifests at multiple levels: from global hull geometry and block assembly, down to local freeform surfaces and functional interfaces of mechanical components. Photogrammetry and laser scanning have been employed for over a decade to reconstruct ship hulls and parts in industrial settings, demonstrating that as-built acquisition can support reengineering tasks that would otherwise require manual surveying. Koelman’s industrial practices-based report already framed photogrammetry-enabled reengineering as a response to recurring needs in ship hull measurement and ship part reconstruction [2]. More recent works have extended the same rationale to large, complex naval structures, showing that combined approaches (e.g., close-range photogrammetry plus terrestrial laser scanning) can mitigate technique-related blind spots and improve coverage and fidelity for ship hull modeling [3].

Parallel to hull-scale applications, the literature documents a growing interest in applying 3D scanning for quantitative quality control during the manufacturing of boats and small craft production, where geometric deviations can accumulate across the mock-up, mold and final part. A representative example proposes high-precision digital shape models from 3D scans to control deviations across key intermediate products in composite small craft production [4]. In a broader shipyard digitalization narrative, these scanning-driven practices align with the idea that dimensional control and traceable geometry are enablers for higher-level digital twin functions, including monitoring and process analysis layers [1].

However, translating these scan-to-model successes into a robust, repeatable workflow is not straightforward, particularly in operational environments where lighting, accessibility, surface condition, vibration and operator variability are intrinsic. This is especially relevant when shipyard reverse engineering (RE) is expected to feed not only documentation but also downstream manufacturing actions, such as additive manufacturing (AM) for rapid spares and replacements.

In addition, the coupling of reverse engineering with additive manufacturing is increasingly framed in the literature as a manufacturing strategy, rather than merely a rapid prototyping convenience, because it enables a scan-to-part route that can shorten lead times, reduce dependence on long spare parts supply chains and support on-demand provisioning when legacy drawings or tooling are unavailable. These conditions are particularly representative of the shipbuilding and ship-refitting sector, which is characterized by small-scale fleets, highly customized components and a pervasive need for rework and retrofit interventions. Early procedural demonstrations already formalized the “optical scanning → digital reconstruction → AM replication” chain [5], while subsequent contributions emphasized that RE outputs often require design intent recovery and, in many cases, redesign for function rather than a naive geometric copy [6,7]. In maritime contexts, where spare parts logistics are particularly cost- and downtime-sensitive, dedicated studies discuss AM-based spare part supply chains and adoption barriers [8]; recent projects and industry-oriented reports synthesize qualification needs, standardization constraints and operational pathways for AM spare parts in the maritime industry (e.g., DAVAMS/SINTEF) [9]. Finally, the metrological dimension remains central: the value proposition of scan-to-AM in maintenance and replacement workflows is only defensible if geometric verification, uncertainty and acceptance criteria are explicit, especially when the part is critical in terms of safety or interface and must pass qualification reasoning rather than qualitative validation.

The integration of RE and AM has been discussed at the methodological level as a route from digitization to physical replication, including template-based approaches for reconstructing mechanical parts from mesh data and converting them into CAD representations usable for fabrication [7,10]. At the same time, the “reverse engineering to shape engineering” perspective emphasizes that the challenge is not only reconstructing geometry, but ensuring that the reconstructed model carries the semantic and functional intent needed for engineering decisions [6,11].

In this context, the metrological credibility of 3D scanning becomes the decisive factor separating demonstration from deployable practice. Range-based and triangulation-based optical sensors can deliver dense surface data rapidly, yet their performance depends on calibration stability, measurement geometry, surface reflectance and algorithmic choices. Foundational work on the performance evaluation of triangulation-based range sensors provides a reference point for understanding how resolution, noise and system characteristics propagate into 3D measurement outcomes [12]. Beyond general performance discussions, dedicated metrology studies have investigated structured-light scanning errors and their contributors. For instance, NIST has reported and analyzed a structured-light 3D scanner, explicitly framing the need for traceability and uncertainty characterization in optical 3D metrology [13,14]. Complementary approaches aim to predict or model structured-light 3D measurement errors in operational conditions [15,16].

A second, frequently underestimated layer is the transition from “raw scan data” to comparative assessment against a reference geometry. In practice, geometric validation often relies on mesh-to-mesh or mesh-to-CAD comparisons, where registration (alignment) is a major driver of the measured deviations. The Iterative Closest Point (ICP) family of methods is widely used, yet it is prone to pitfalls that can materially bias deviation maps and summary metrics. Decker et al. explicitly analyzed potential pitfalls of ICP-based registration in the shape accuracy assessment of additively manufactured parts, showing that registration errors can be large enough to distort conclusions [17,18]. Along the same axis, research has proposed calibration and correction strategies to address scanner-specific distortions, such as mapping and correcting structured-light scanner distortion fields as a route to improved accuracy [17,19].

Crucially, a robust assessment requires moving from deviation visualization to a decision-oriented interpretation. In this respect, it is useful to note that the broader “quality assessment” community has recently converged toward survey-level consolidation of evaluation paradigms, stressing the need for versatile, multi-indicator frameworks rather than single-score metrics, particularly when heterogeneous data, diverse operating conditions, and deployment constraints must be jointly accounted for. For example, recent overviews emphasize how modern evaluation pipelines increasingly combine complementary indicators, benchmark ecosystems, and unified assessment protocols to improve robustness and comparability across tasks and modalities [20,21].

In general, 3D scan deployment in shipyards depends on practical, repeatable indicators that jointly capture geometric completeness, global agreement with a reference model, and dimensional consistency over critical features that matter for function and assembly. In this sense, assessment procedures should make explicit not only “how far” a scan deviates from nominal geometry, but also whether deviations are spatially localized, whether critical dimensions remain stable across repeated regions, and whether the resulting reconstruction is fit for the intended reverse engineering task.

The shipyard domain adds an additional layer of complexity: the same geometric pipeline must often operate under time pressure, limited fixturing, and sub-ideal measurement setups, while still producing output that is fit for downstream actions, potentially including manufacturing. This raises practical questions: when does a scan become good enough to support reverse engineering and potentially physical replication? Under which constraints can this be asserted credibly? The literature offers multiple partial answers: ship hull and block-level digitization for documentation and verification; quality control use cases; theoretical frameworks for integrating RE with AM [2,4]. However, end-to-end workflows that explicitly couple shipyard-realistic acquisition, registration-aware validation and quantitative features relevant quality indicators, while also considering the downstream implications for additive reproduction, remain limited, particularly when portable field-oriented scanners and rapid manufacturing routes are involved.

This gap is particularly relevant when the objective is not solely to create a digital artifact, but to enable sustainable operational practices. Shipyards and maritime operators are increasingly interested in reducing waste and lead times in spare parts provisioning and maintenance logistics; AM is often discussed as an enabler of on-demand parts and reduced inventory burden, but the viability of this vision depends on dimensional assurance at the point of use. Accordingly, the present work presents the development and the experimental results of a quantitative scan to reference a quality-based comparison framework based on three independent indicators analyzing scan completeness, global geometric agreement and dimensional fidelity in terms of measurement accuracy and variability. The proposed workflow and experimental analysis are aimed at supporting 3D scan and reverse engineering diffusion in shipyard environments, and the indicators are defined to quantitatively support rapid scan acceptance/rejection decisions based on reproducible criteria, rather than unique metrological indicators. The approach was demonstrated on a purpose-designed mechanical specimen manufactured in both reflective metal and low-reflectivity polymer and used to compare a field-oriented portable scanner and a metrology-oriented system under representative surface conditions and tracking enhancement strategies.

The research was conducted as part of the SHIPLEARNING (Shipbuilding innovation through introduction of 3D scanning and machine-learning-assisted FSW processes) project, funded by NextGenerationEU, National Recovery and Resilience Plan, Mission 4, Component 2, Investment 1.5, on the research program of “iNEST—Interconnected Nord-Est Innovation Ecosystem” Innovation Ecosystem Consortium, Spoke 5 “Smart and Sustainable Environments (Manufacturing, Working, Living)”.

## 2. Materials and Methods

### 2.1. Mechanical Components

**Figure 1:**
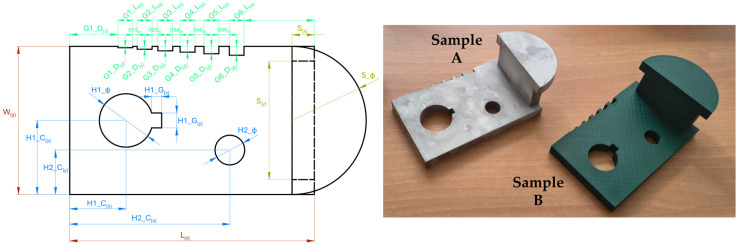
Mechanical sample designed for quantitative scan comparison. (**Left**) CAD model (top view), with indication of the geometrical features, colored according to the feature type: main (red), holes (blue), grooves (green). (**Right**) manufactured samples in metal (A—high surface reflectivity) and in PLA (B—low surface reflectivity).

To experimentally compare 3D scanning performance with different acquisition systems and surface conditions representative of real-world shipyard applications, a purpose-made mechanical sample was designed. The geometry, reported in Figure 1 (left), was defined to replicate the complexity and constraints typically encountered in mechanical components used in shipbuilding, allowing a consistent dimensional analysis across different scanning and production methods. The sample, with overall dimensions of 200 mm (length), 100 mm (width) and 65 mm (height), includes internal cavities, sharp edges, inclined surfaces and regions with variable curvature. These features are known to challenge 3D scanning and subsequent geometry reconstruction. To investigate the influence of material and surface conditions on the reverse engineering process, two samples with similar nominal geometry were manufactured using different processes. Sample A was produced in metal using conventional computerized numerical control (CNC) machining, a widely adopted production process in shipyards. Sample B was produced in polylactic acid (PLA) using fused filament fabrication (FFF) technology, representing an innovative manufacturing process for rapid prototyping. A X1 Carbon 3D printer (Bambulab, Shenzhen, China) was employed. The two samples are shown in Figure 1 (right).

This dual manufacturing allowed a direct and robust comparison between traditional and additive manufacturing approaches under controlled geometric conditions. In order to provide a reliable reference for 3D scan quality assessment from both geometrical and dimensional aspects, the manufactured samples were geometrically characterized by direct measuring using a caliper with 0.05 mm resolution. The resulting detailed dimensions, calculated as the mean value over three independent measures, are reported in Table 1. This geometrical characterization was used to generate sample-specific reference CAD models, useful for subsequent scan vs. CAD comparisons, thereby filtering potential differences due to manufacturing deviations from the nominal design, and allowing the evaluation of the scan quality with respect to the actual manufactured geometry.

### 2.2. Scanning 3D

Two 3D scanning systems were employed to evaluate the performance of reverse engineering workflows under conditions representative of shipyard applications. Both systems were manufactured by Revopoint (Shenzhen, China), and differ substantially in sensing principle, operational workflow and potential metrological performance.

The MIRACO NIR is a portable structured-light scanner designed as a standalone unit, integrating four depth cameras and a 48 MP RGB camera, together with on-board data computing and storage (octa-core processor, 32 GB of RAM and 256 GB of storage). A 6-inch 2K AMOLED touchscreen allows field acquisition, inspection and preliminary editing without the need for an external workstation, making the device particularly suitable for constrained environments, such as shipyards. However, using infrared structured-light projection, the system may suffer with highly reflective metallic surfaces, thus requiring surface preparation (scanning sprays) or optical markers to improve tracking stability and data reliability.

The METRO X is a metrology-oriented system integrating multiple scanning technologies, such as 14 crossed blue laser lines, 7 parallel blue lines and 62 full-field structured-light lines, dynamically adapting to the geometry and surface properties of the target object. The system supports multiple scanning modes (manual, tripod-based, or with a rotating platform) and tracking strategies (feature- and marker-based), which give strong advantages in the case of complex geometries requiring reliable registration and global alignment. Therefore, the system provides increased robustness and accuracy with industrial parts, including untreated metallic surfaces. This higher metrological performance directly affects the system portability, strongly reducing it due to the need for an external computing workstation equipped with a dedicated GPU for acquisition and processing. Moreover, the increased operational complexity leads to the necessity of trained operators. The main specifications of both devices are summarized in Table 2.

In the context of small shipyards, where scanning is frequently performed on-site on machined or untreated metallic components with limited environmental control, the scan quality and repeatability are strongly influenced by surface reflectivity, geometric complexity, and accessibility and operational constraints.

Surface reflectivity remains a critical factor, as highly reflective surfaces can scatter or reflect the projected light, leading to increased noise, local loss of data and geometric inaccuracies. Infrared structured-light systems such as MIRACO NIR are typically more sensitive to these effects; however, laser-based solutions such as METRO X may also show a performance reduction with highly reflective regions. In practice, both scanning systems can benefit from surface preparation using scanning sprays or attaching optical markers, which help in stabilizing the acquisition process.

Target component geometric complexity and accessibility may also affect scanning performance, especially in the presence of deep cavities, narrow slots and sharp edges, resulting in potential tracking loss and local point density decrements. The METRO X system can exploit dynamic scanning modes, making it more suitable for the precision scanning of small, detailed parts in controlled settings. Conversely, the compactness and standalone operation of the MIRACO NIR facilitate rapid acquisition in restricted spaces and in time-constrained field operations.

The experimental acquisitions of the mechanical components manufactured in metal and PLA were performed using the two scanning systems. During the acquisition, the components were positioned on a flat surface, and two independent scans were conducted to reconstruct both sides. The two resulting point clouds were then merged to obtain a single cloud for each component with different scanning conditions.

**Figure 2:**
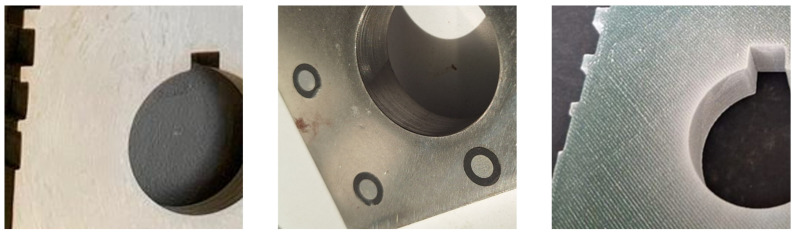
Evaluation of scanning performances under different surface scenarios. Example of the tested condition for the metal sample: raw surface without treatments (**left**), optical tracking markers (**center**), temporary matte scanning spray (**right**).

In order to evaluate the scanning performances under different surface conditions, the acquisitions were performed using three different scenarios, reported in Figure 2, namely: (i) raw, with the material as-is after the manufacturing process, (ii) marker, attaching circular (*D_in_* = 3 mm–*D_ext_* = 6 mm) optical markers (Revopoint) useful to help the scan tracking, (iii) spray, using a temporary scanning paint (AESUB blue, Revopoint) useful to stabilize the acquisition process.

### 2.3. Data Processing Algorithm

For each component scan, seven planar sections were extracted from the point clouds to evaluate the geometric correspondence between the acquired data and the reference component shape. All sections were taken at constant spacing to cover the entire thickness of the lower face of the component, which is the region richest in functionally relevant dimensional features. Each section was represented by the nominal CAD contour and by the corresponding profile reconstructed from the 3D scan.

**Figure 3:**
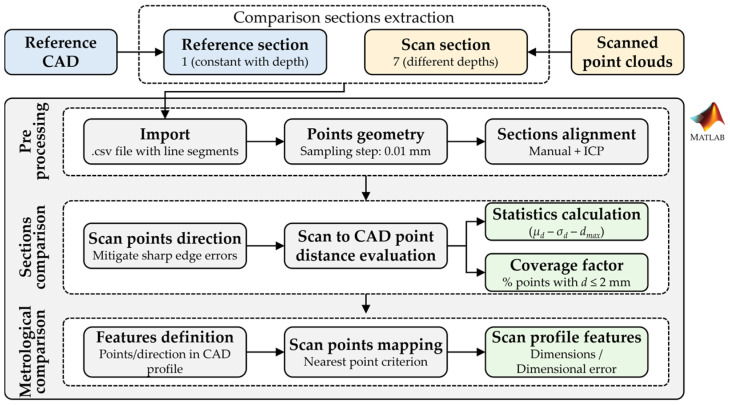
Workflow schematization of the algorithm implemented in MATLAB for the processing and comparison of the reference and scanned sections: sections extraction (1 reference + 7 scanned), preprocessing (point geometries and alignment), sections comparison (distance evaluation, coverage factor and statistical distributions), geometrical features dimensions computation.

A customized processing algorithm was implemented in MATLAB R2024b (MathWorks, Natick, MA, USA) to analyze the planar sections and extract relevant data useful for scan quality assessment, following the workflow reported in Figure 3. The algorithm was designed to: (i) register the scanned sections in a common frame with the CAD model, (ii) quantify pointwise geometric deviations between the measured and nominal profiles, (iii) derive a set of dimensional features relevant for subsequent scan quality assessment.

Both the reference and the scanned sections were composed of chains of line segments, imported in MATLAB from .csv files. To allow a complete geometrical comparison, a dense point representation of each profile was reconstructed by resampling all segments with a fixed step of 0.01 mm, small enough with respect to the characteristic dimensions of the inspected features. The resampled profiles were thus reshaped as a cloud of points.

After this, the sections alignment process was carried out following two sequential steps. Firstly, a rigid translation of the scanned section was applied by selecting two pairs of homologous points on the scanned and CAD profiles, constraining the horizontal and vertical alignments. The corresponding translation vectors were applied to all scan points, moving the scanned section in the CAD reference frame. Secondly, the alignment was refined by means of an iterative closest point (ICP) procedure, which minimizes the distance between pairs of closest points on the two profiles. As all sections were initially extracted with a consistent orientation, rotational misalignment could be assumed negligible and no rotations were applied.

Geometric deviations between the measured and reference profiles were first quantified by computing the Euclidean distance between each scan point and its homologous reference point, identified as the nearest neighbor on the CAD contour. For each point, both unsigned (*d_un_*, absolute) and signed (*d_s_*) distances were computed to evaluate a general scan quality parameter and to distinguish material excess from material deficit.

A refinement of the methodology was introduced in regions characterized by sharp rectangular features. Indeed, in the presence of sharp angles, the homologous point definition based on the nearest neighbor criterion alone can lead to incorrect associations, as scan points close to a corner may be incorrectly matched to CAD points belonging to the adjacent segment. To mitigate this effect, the local orientation of the profile at each scan point was estimated by linear fitting using the four nearest neighbors and computing the corresponding angle (α) with respect to the horizontal axis. An angular threshold was defined to classify each point as predominantly horizontal (α < 15°) or vertical (α > 15°), thus guiding the search of the homologous CAD point. For points classified as horizontal, the homologous CAD point was searched among those with similar *X* coordinates, minimizing the distance along *Y*; for points classified as vertical, the search was restricted to CAD points with similar *Y* coordinates, minimizing the distance along *X*.

For each scanned section, the global statistics of the unsigned distance to the reference profile were then computed. In particular, the mean distance (*µ*_*d*,*un*_), the standard deviation (*σ*_*d*,*un*_), and the maximum deviation (*d*_*un*,*max*_) over all points were evaluated at section level. These quantities provide a compact description of the agreement between the measured section and the nominal CAD contour.

**Figure 4:**
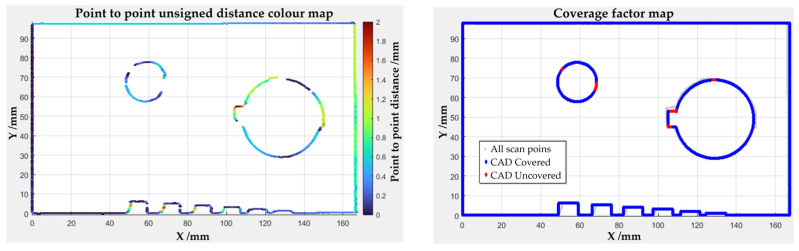
Example of algorithm outputs regarding the scan geometry correspondence with the reference model. (**Left**) scan to reference point to point unsigned distance color map, ranging from 0 mm (dark blue) to 2 mm (dark red). (**Right**) Coverage Factor map reporting the reference points “covered” (blue) and the “uncovered” (red), based on a minimum distance threshold (*R* = 2 mm).

Starting from the unsigned distance data, a section-level Coverage Factor (*CF_s%_*) was then defined as the percentage of the CAD reference profile points that are effectively represented in the scan profile. Each point of the reference profile was classified as “covered” or “uncovered” by comparing its distance to the nearest homologous point in the scan profile with a threshold (*R* = 2 mm); points with a distance less than or equal to the threshold were considered covered. The overall section Coverage Factor was defined as:

(1) $${CF}_{s\%}=\frac{100}{N_{ref}}\cdot \sum_{i=1}^{N_{ref}}I(d_{un,i}\leq R)$$

where *N_ref_* is the number of points composing the CAD profile, *d*_*un*,*i*_ is the unsigned distance from the i-th CAD point to the nearest homologous scan point, *I* is the indicator function, equal to 1 for covered points or 0 for uncovered points. The *CF_s%_* can describe how completely the nominal CAD profile is represented by the scanned data at section level. An example of the algorithm outputs is reported in Figure 4 in terms of point-to-point unsigned distance color map and Coverage factor map.

**Figure 5:**
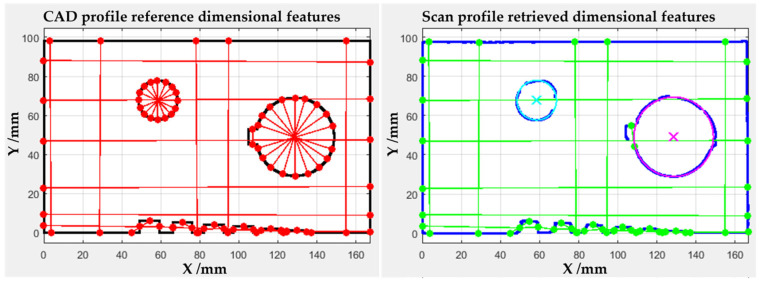
Example of algorithm outputs regarding the scan measurement correspondence with reference model. (**Left**) points selected in the reference CAD section to derive geometrical features (in red) nominal dimension (Table 1). (**Right**) homologous points mapped in the scan section based on nearest point criterion to derive scanned geometrical features (in green) dimension. The crosses indicate the holes centers estimated by least- squares circle fitting.

After this, the algorithm automatically derived a set of dimensional features used to evaluate the measurement correspondence between the reference CAD and the scanned sections. A set of dimensions of interest was selected on the reference section by defining appropriate reference points, as illustrated in Figure 5. These points were then mapped into the scanned section using the nearest point criterion, thereby identifying the corresponding scan points to be used for dimensional evaluation.

Each feature was defined by two reference points in the CAD section and a predefined direction (*X*, *Y*, *Random*), which specified how the corresponding scan dimension had to be computed. For features with a prescribed direction (*X*, *Y*), the scan dimension was calculated as the absolute difference between the coordinates of the two homologous points along the selected axis, in order to avoid errors due to residual misalignment in the undesired direction. For features with unspecified direction (*Random*), the scan dimension was calculated as the Euclidean distance between the two homologous scan points. This latter approach was used in particular for the two circular holes, whose centers and mean diameters were estimated by a least-squares circle fitting of the scan points identified on the circumferences. The resulting features were compared with those obtained by averaging the different diameters estimated with the Euclidean distance method.

All the information produced by the algorithm formed the basis for the scan level quality indicators described in the following subsection.

### 2.4. Quantitative Scan Comparison

The information computed from the seven individual sections extracted from the scan point cloud were aggregated to characterize the global quality of each 3D scan. Three complementary metrics were defined: (i) the scan level Coverage Factor (*CF*_3*D*%_), quantifying geometric completeness; (ii) the Mean Distance (*MD*_3*D*_) between the scan and the CAD model, describing global geometrical agreement; (iii) the Scan Quality index (*SQI*_3*D*_) linking scan quality to functionally relevant dimensions and their tolerances. Taken together, these parameters offer a comprehensive description of the overall scan quality, as they jointly account for geometric completeness, agreement with the reference model and measurements compliance. They provide a quantitative basis for comparing scans and acquisition strategies in relation to the functionality requirements of the component.

**Coverage Factor (*CF*_3*D*%_)**

The Coverage Factor (*CF*_3*D*%_) was defined to quantify the geometric completeness of the reconstructed scan with respect to the reference model. It computes the percentage of CAD reference points effectively represented in the scan within a predefined coverage distance threshold, thereby providing a direct indicator of missing or poorly reconstructed regions. Its definition is aligned with threshold-based completeness and coverage metrics used in reference-to-scan validation [22]. The Coverage Factor at section level (*CF_s%_*) was already defined in Equation (1). For each 3D scan, a scan level Coverage Factor (*CF*_3*D*%_) was obtained by averaging the seven section level coverage values, while the corresponding standard deviation (*σ_CF_*_3*D*%_) quantifies its variability over the sample depth:

(2) $${CF}_{3D\%}=\frac{1}{7}\cdot \sum_{s=1}^{7}{CF}_{s\%,s}$$

A high *CF*_3*D*%_ indicates that the scanning system effectively sampled the entire nominal contour within the prescribed threshold, while a low *CF*_3*D*%_ reveals the presence of unsampled or poorly represented regions. In addition, a large standard deviation highlights inhomogeneity in the scan completeness among the different sections, while a low variability suggests uniformly reproduced geometry at 3D level.

**Mean Cloud-to-CAD Distance (*MD*_3*D*_)**

The Mean Cloud-to-CAD Distance (*MD*_3*D*_) was adopted as a compact quantitative descriptor of the global geometric agreement between the scanned point cloud and the CAD model. It was computed from the closest homologous point distances and aggregated across the seven planar sections. When used together with its standard deviation (*σ_MD_*_3*D*_), it captures both the overall geometric deviation magnitude and its non-uniformity along the sample thickness. The *MD*_3*D*_ definition is derived from the C2C (Cloud to Cloud) and C2M (Cloud to Model) distance, normally implemented in all the 3D processing softwares [23,24], with the main difference being that distances are evaluated on consistently extracted planar sections and then averaged, thereby allowing direct comparison and repeatability assessment across sample thickness. For each 3D scan, a scan level Mean Distance (*MD*_3*D*_) was calculated by averaging the seven section level mean unsigned distances (*µ*_*d*,*un*_), while the corresponding standard deviation (*σ_MD_*_3*D*_) quantified its variability over the sample depth.

(3) $${MD}_{3D}=\frac{1}{7}\cdot \sum_{s=1}^{7}\mu_{d,un,s}$$

The *MD*_3*D*_ parameter captures the overall magnitude of the geometric deviation between scan and CAD: low values indicate high geometrical conformity with respect to the reference sample, whereas high values denote poor geometrical agreement. The *σ_MD_*_3*D*_ provides insight into how uniformly this distance is distributed along the component, revealing potential local scan issues.

**Scan Quality Index (*SQI*_3*D*_)**

A third parameter, the Scan Quality Index (*SQI*_3*D*_) was defined to complement the *CF*_3*D*%_ and *MD*_3*D*_ with an operational and requirement driven indicator customized to a reverse engineering perspective. Indeed, it is computed from the set of functionally relevant dimensional features defined in the previous section (Table 1 and Figure 5), condensing the measurement bias and variability into a normalized feature score, weighted by feature-related geometric tolerances to reflect different functional criticalities. In this way, the *SQI*_3*D*_ is intended to quantitatively support rapid scan acceptance/rejection decisions based on dimensional requirements and reproducible criteria, rather than providing a single metrological accuracy indication.

For each feature (*j*), the nominal value (*L*_*REF*,*j*_) was retrieved from the CAD definition. The processing of the seven sections extracted for each 3D scan provided seven measured values for the same feature, from which the mean value (*L*_3*D*,*j*_) and the standard deviation (*σ*_3*D*,*j*_) were computed at scan level. To account for both systematic and random contributions, a dimensional error (*Err*_3*D*,*j*_) was defined for each feature by combining the average deviation, calculated as the difference between the scan-based mean value and the CAD reference dimension, and the standard deviation (*σ*_3*D*_) over the seven sections:

(4) $${Err}_{3D,j}=\sqrt{{\left|L_{3D,j}-L_{REF,j}\right|}^{2}+\sigma_{3D,j}^{2}}$$

In order to use the *SQI*_3*D*_ as a fast operation decision support, its definition explicitly accounts for feature-dependent dimensional tolerances to reflect the different functional criticalities. Consequently, the parameter can be tuned to the specific operational requirements. A relative tolerance level (*τ_j_*), expressed as a percentage of the nominal dimension, was then assigned to each feature. In the deterministic case used for the experimental work, the tolerance assignment was driven by the question: “in a reverse engineering process, what is the impact of a potential scan error on this specific dimension?”. According to this criterion, functionally critical features, such as holes and main external dimensions, were assigned tighter tolerances (e.g., *τ_j_* = 1%), while small or sub-critical features, such as groove thicknesses, were assigned larger tolerances (e.g., *τ_j_* = 5%). The corresponding absolute tolerance (*T_j_*) was obtained by multiplying with the nominal dimension. For each measured feature, a normalized scan score (*S_j_*) was then computed as:

(5) $$S_{j}=\frac{1}{1+{Err}_{3D,j}/T_{j}}$$

In the ideal case of perfect agreement between the estimated dimension from the scan and the nominal value (*L*_3*D*,*j*_ = *L*_*REF*,*j*_) and absence of variability among the seven sections (*σ*_3*D*,*j*_ = 0), a *S_j_* = 1 will be assigned to the *j*-th feature, indicating that the scan is fully capable of reproducing that feature. As either the dimensional error or the assigned tolerance becomes less favorable, the parameter Sj tends to decrease. As an example, *S_j_* = 0.5 will be assigned when the dimensional error equals the assigned tolerance (*Err*_3*D*,*j*_ = *T_j_*).

The normalized scores evaluated for all the selected features were then averaged to define a global scan quality index (*SQI*_3*D*_), characteristic of the measurement correspondence between the scanned shape and the reference model.

By construction, the *SQI*_3*D*_ ranges between 0 and 100 and provides a scalar indicator of the scan quality directly related to the dimensional requirements of the component. In practice, values of *SQI*_3*D*_ close to 100 correspond to scans in which all the key dimensions are well reproduced within their assigned tolerances, whereas values significantly below indicate that several functionally relevant features exceed their tolerance levels.

## 3. Results

The present study aims at evaluating the potential application of reverse engineering processes in shipyard environments, by comparing the performance of two scanning systems (MIRACO NIR and METRO X) used on reflective metallic and non-reflective PLA samples under different surface conditions (raw, marker, spray).

In the next sections, the obtained results are presented from both qualitative and quantitative points of view.

### 3.1. Qualitative Scan Comparison

**Figure 6:**
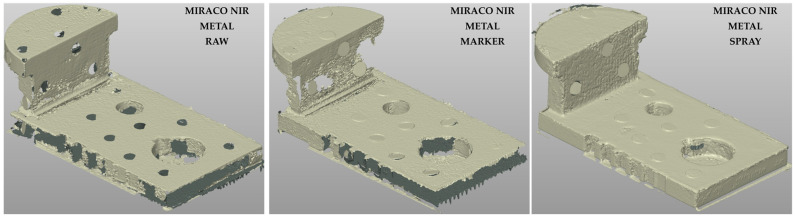
3D point clouds computed with MIRACO NIR system for the Metal sample (high surface reflectivity) with different surface conditions: raw surface without treatments (**left**), optical tracking markers (**center**), temporary matte scanning spray (**right**).

The point clouds reconstructed with the MIRACO NIR on the metal sample are reported in Figure 6 for the three surface conditions: raw (left), marker (center), spray (right).

The visual results suggest that under raw surface condition (Figure 6, left), the geometry reconstruction shows several criticalities, with pronounced discontinuities and local loss of data, consistent with issues in scan tracking on high reflectivity surfaces. These artifacts are more evident on small lateral faces and in regions with sharp angle variations, where rapid changes in surface orientation aggravate tracking stability. As a consequence, while the main external dimensions are normally represented, small scale geometrical features, such as grooves, are poorly captured or not present at all.

Marker-assisted acquisition (Figure 6, center) improves the overall quality of the reconstructed point cloud compared to the raw scenario. However, the sensitivity to small features remains limited, with grooves still not reproduced. This behavior can be attributed to the marker size, which constrains the placement in correspondence to small features and sharp corners, thus reducing the tracking enhancement in the most challenging regions. In addition, when markers are physically attached to the sample surface, their presence introduces local perturbations that appear in the reconstructed point cloud as circular thicker areas. In the present dataset, circular artifacts can be recognized on all the reconstructed clouds; also in the raw acquisition, where markers were physically present but marker-based tracking was disabled, circular holes can be observed where markers were present, leading to local data loss in those regions.

The use of scanning spray (Figure 6, right) led to the most consistent results in terms of geometrical continuity and sensitivity to small features. The possibility of covering the entire sample surface with the spray improves tracking robustness across sharp transitions and small lateral surfaces as well, resulting in a more homogeneous point cloud in which grooves and other small details are normally detected. Also, in this case, circular thicker areas remain locally visible where markers were attached, even if the marker tracking mode was disabled, due to the local surface alteration induced. However, this artifact does not affect the results related to the quality through the three independent parameters.

**Figure 7:**
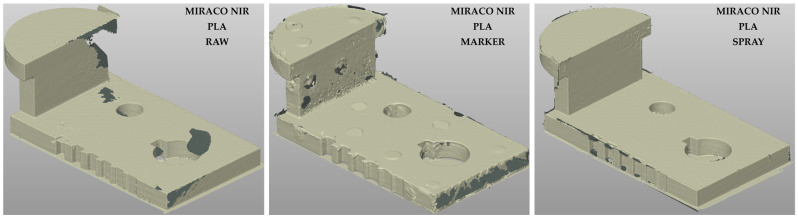
3D point clouds obtained with MIRACO NIR system for the PLA sample (low surface reflectivity) under different surface conditions: raw surface without treatments (**left**), optical tracking markers (**center**), temporary matte scanning spray (**right**).

The point clouds reconstructed using the MIRACO NIR system on the PLA sample are shown in Figure 7 for the same surface conditions. Compared to the metal sample, a clear qualitative improvement is observed across all conditions, confirming the strong impact of surface reflectivity on reconstruction quality. Under raw conditions (Figure 7, left), the point cloud appears quite continuous, with the small features generally captured, although limited areas with reduced tracking quality can still be identified. Unlike the metal sample, marker-assisted acquisition (Figure 7, center) does not provide substantial improvements for the low-reflectivity PLA surface, whereas the use of scanning spray (Figure 7, right) further improves homogeneity and reduces local artifacts, remaining the most effective strategy for scan-tracking enhancement among the tested conditions.

**Figure 8:**
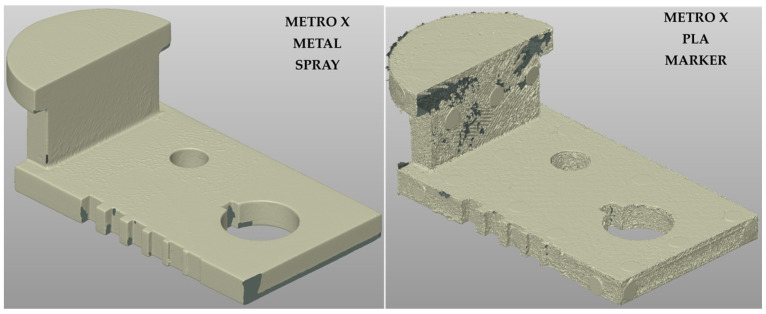
3D point clouds obtained with METRO X system for the metal sample with temporary matte scanning spray (**left**) and PLA sample with optical tracking markers (**right**).

For the METRO X system, the high surface reflectivity of the metal sample proved to be a critical factor. Indeed, in the tested raw and marker assisted conditions, consistent geometrical reconstruction could not be obtained under the tested settings. Only the use of scanning spray enabled the generation of a complete and stable point cloud (Figure 8, left), characterized by high continuity and homogeneity, capturing all the small geometrical features. Qualitatively, the METRO X combined with scanning spray provided the best 3D reconstruction of the metal sample among the tested configurations.

For the PLA sample, the METRO X system produced a high-quality 3D reconstruction under marker-assisted acquisition (Figure 8, right), showing improved homogeneity compared with the MIRACO NIR under the same condition (Figure 7, center). This result is consistent with the lower impact of reflectivity of PLA surface, for which both tested systems can achieve good results, with the metrology-oriented system offering higher reconstruction quality.

### 3.2. Quantitative Scan Comparison

Quantitative scan comparison was performed through the three scan level parameters derived from the seven extracted sections described in Section 2.3: Coverage Factor (*CF*_3*D*%_), Mean Cloud-to-CAD Distance (*MD*_3*D*_), and scan quality index (*SQI*_3*D*_). The estimated parameters for all tested samples and conditions are reported in Table 3, and discussed in detail in the next Figures, which present them as mean values calculated over the seven extracted sections, with error bars representing the standard deviation across sections, which quantifies the variability of the parameter along the sample thickness.

**Figure 9:**
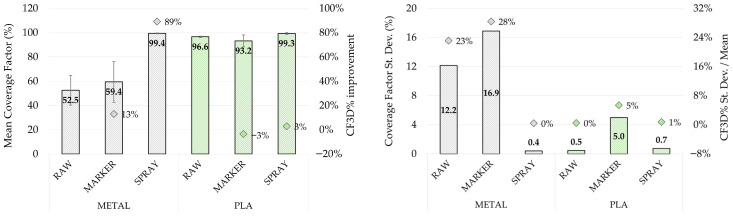
Coverage Factor (*CF*_3*D*%_) evaluated for each reconstructed point cloud obtained by MIRACO NIR. (**Left**) mean value comparison between acquisitions on metal sample (black) and PLA sample (green) under different surface conditions, with error bars representing the standard deviation across sections and dots indicating the increment with respect to raw surface conditions. (**Right**) absolute standard deviation (bars) and mean normalized standard deviation (dots) computed across the seven horizontal sections, indicating the parameter variation along the sample thickness.

Figure 9 reports the estimated *CF*_3*D*%_ for both metal and PLA samples under the tested surface conditions (raw, marker, spray) obtained with MIRACO NIR scanning system. Results are reported in terms of mean value (left) and standard deviation (right) computed over the seven extracted horizontal sections. For the metal sample, the raw surface scenario showed a limited *CF*_3*D*%_ value (52.5 ± 12.2%), which slightly increased with marker-assisted acquisition (59.4 ± 16.9%, +13%). Considering that the Coverage Factor ranges between 0 and 100, indicating the percentage of the reference section points correctly mapped in the scanned section, these results show that under these scanning conditions less than 60% of the geometry is reproduced. These outcomes are consistent with qualitative inspection, which highlighted geometry discontinuities and local loss of data due to scan-tracking instabilities on reflective surfaces. In addition, the same conditions showed higher parameter standard deviation with respect to the average value (23% and 28% respectively) compared to the other conditions, indicating a high variability along the sample thickness with a consequent non-uniform reconstruction.

A strong *CF*_3*D*%_ improvement (+89% with respect to raw surface) was achieved using scanning spray, resulting in an almost complete and highly homogeneous scan coverage (99.4 ± 0.4%), in line with the increased reconstruction continuity qualitatively observed in Figure 6. For the PLA sample, the *CF*_3*D*%_ tended to be generally high (above 93%) in all tested conditions, indicating that for low-reflectivity surfaces the scan completeness is generally less critical and less sensitive to tracking enhancement methods. In particular, the use of tracking markers resulted in a lower factor value and higher variability along the thickness (93.2 ± 5.0%), highlighting worsening scan conditions. Contrarily, for PLA the highest coverage was also obtained with scanning spray (99.3 ± 0.7%).

**Figure 10:**
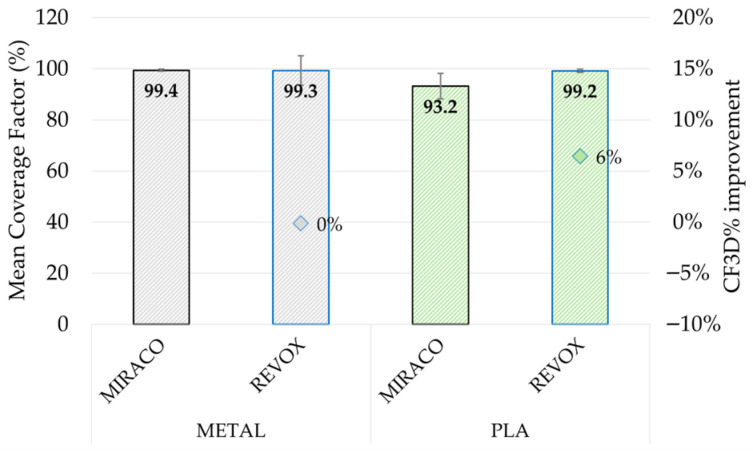
Coverage Factor (*CF*_3*D*%_): mean value comparison between acquisition performed with MIRACO NIR (black-bordered bars) and REVO X (blue-bordered bars) on metal sample (gray-colored bars) with scanning spray and PLA sample (green-colored bars) with tracking markers. The error bars represent the standard deviation across sections.

The scanner comparison results are reported in Figure 10 in terms of *CF*_3*D*%_ mean value computed over the seven extracted horizontal sections, with error bars showing the parameter variability. Under comparable conditions (metal sample with scanning spray and PLA sample with tracking markers), both systems achieved generally high Coverage Factors (*CF*_3*D*%_ = 93.2 ÷ 99.4%), with only a limited increment (+6%) for REVO X in the PLA sample. This result confirms that also in high-surface-reflectivity scenarios, when an effective tracking strategy is implemented, continuous 3D reconstruction can also be obtained with portable systems that are not primarily metrology-oriented.

**Figure 11:**
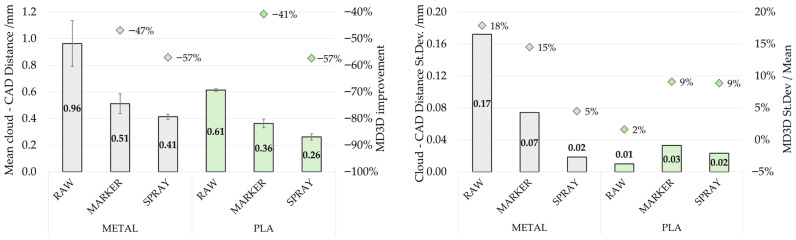
Cloud to CAD Distance (*MD*_3*D*_) evaluated for each reconstructed point cloud obtained by MIRACO NIR. (**Left**) mean value comparison between acquisitions on metal sample (black) and PLA sample (green) under different surface conditions, with error bars representing the standard deviation across sections and dots indicating the reduction with respect to raw surface conditions. (**Right**) absolute standard deviation (bars) and mean normalized standard deviation (dots) computed across the seven horizontal sections, indicating the parameter variation along the sample thickness.

Figure 11 reports the estimated *MD*_3*D*_ for both metal and PLA samples under the tested surface conditions (raw, marker, spray) obtained with MIRACO NIR scanning system. Results are reported in terms of mean value (left) and standard deviation (right) computed over the seven extracted horizontal sections. For the metal sample, the raw surface scenario led to the largest parameter value (0.96 ± 0.17 mm), which decreased using surface treatment strategies: markers (0.51 ± 0.07 mm, −47%) and scanning spray (0.41 ± 0.02 mm, −57% with respect to raw surface). Considering that the cloud to CAD distance indicates the correspondence of the geometrical reconstruction, a lower value is obtained only if the scanned sections overlap well with the reference section. This trend confirms the effectiveness of surface preparation and tracking enhancement methods on reflective materials. In addition, the *MD*_3*D*_ standard deviation tends to progressively reduce from raw (0.17 mm) to marker (0.07 mm) and spray conditions (0.02 mm), highlighting that a more homogeneous geometry reconstruction along sample thickness is achieved with a tracking strategy on high reflectivity materials. A similar trend, with progressive reduction from raw to marker and spray conditions, can be observed for the PLA sample as well, which however exhibited systematically lower *MD*_3*D*_ mean value than the metal sample under comparable conditions (−36% for raw surface, −29% for markers, −37% for spray) also with lower standard deviation, confirming that surface reflectivity and acquisition stability strongly influence geometric reconstruction.

**Figure 12:**
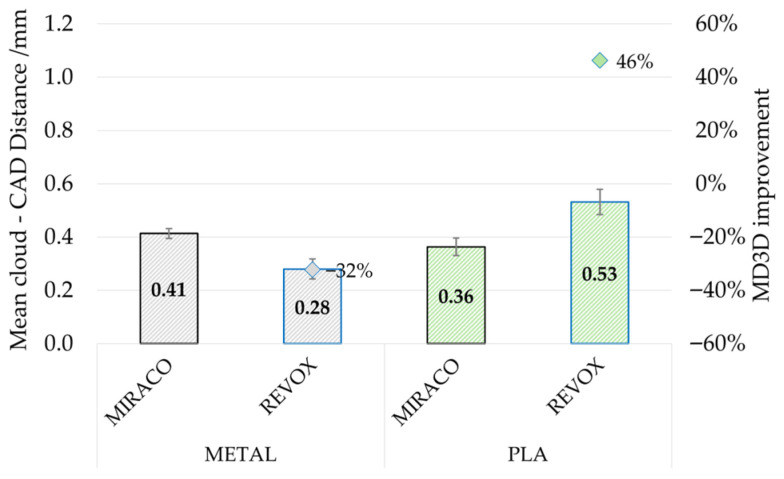
Cloud to CAD Distance (*MD*_3*D*_): mean value comparison between acquisition performed with MIRACO NIR (black-bordered bars) and REVO X (blue-bordered bars) on metal sample (gray-colored bars) with scanning spray and PLA sample (green-colored bars) with tracking markers. The error bars represent the standard deviation across sections.

The scanner comparison results are reported in Figure 12 in terms of *MD*_3*D*_ mean value computed over the seven extracted horizontal sections, with error bars showing the parameter variability. Under comparable conditions (metal sample with scanning spray and PLA sample with tracking markers), a different scanning system impact can be noted depending on the material. In the case of the metal sample, REVO X metrology-oriented system resulted in a marked reduction in *MD*_3*D*_ (0.28 ± 0.04 mm, −32%) with respect to the portable MIRACO NIR. Conversely, for the PLA sample, REVO X resulted in a higher *MD*_3*D*_ (0.53 ± 0.05 mm, +46%) with respect to MIRACO NIR.

The *SQI*_3*D*_ parameter accounts for the measurement accuracy of the scanned geometry based on the predefined set of dimensional features, combining the mean bias with respect to the nominal value and the standard deviation across the sections. The geometrical features’ dimensional characterization obtained from each reconstructed point cloud are reported in terms of mean value and standard deviation in Table 4 for the metal sample and in Table 5 for the PLA sample.

**Figure 13:**
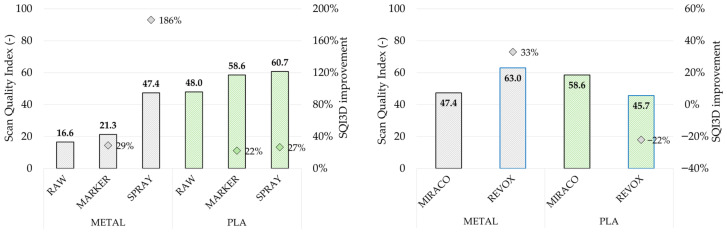
Scan Quality Index (*SQI*_3*D*_) evaluated for each reconstructed point cloud obtained by MIRACO NIR. (**Left**) comparison between acquisitions on metal sample (black) and PLA sample (green) under different surface conditions, with dots indicating the increment with respect to raw surface conditions. (**Right**) comparison between acquisition performed with MIRACO NIR (black bordered bars) and REVO X (blue-bordered bars) on metal sample (gray-colored bars) with scanning spray and PLA sample (green-colored bars) with tracking markers.

Assuming the deterministic tolerance scenario defined in Section 2.4, the calculated *SQI*_3*D*_ values are shown in Figure 13 (left) for both the metal and PLA samples under the tested surface conditions (raw, marker, spray) obtained with MIRACO NIR scanning system. For the metal sample, the raw surface scenario showed a very low *SQI*_3*D*_ value (16.6), which slightly increased with marker-assisted acquisition (21.3, +29%). Considering that the scan quality index ranges between 0 and 100, these values indicate a poor reliability of the reconstructed geometry, with a consequent limited suitability for reverse engineering tasks requiring dimensional consistency. Scanning spray led to a substantial improvement of *SQI*_3*D*_ (47.4, +186% with respect to raw surface), consistent with the trend observed for the other parameters, confirming this as the most reliable tracking enhancement method, as well as from a measurement perspective. These outcomes, consistent with the trends observed for the other parameters (*CF*_3*D*%_ and *MD*_3*D*_), highlight that correct surface preparation is therefore an essential step not only to increase point cloud homogeneity and completeness, but also to achieve dimensional accuracy on functionally relevant features.

A similar trend, with progressive increment from raw to marker and spray conditions, can be observed for the PLA sample as well, however, which exhibited systematically higher *SQI*_3*D*_ values than the metal sample under comparable conditions (+190% for raw surface, +174% for markers, +28% for spray), consistently with the reduced impact of reflectivity on both reconstruction stability and dimensional reliability. For this sample, the *SQI*_3*D*_ increase with tracking enhancement strategies remained, but limited with respect to metal (+22% with marker, +27% with spray), due to already favorable low-reflectivity raw surface conditions.

Figure 13 (Right) reports the scanner comparison results. Under comparable conditions (metal sample with scanning spray and PLA sample with tracking markers), a coherent *SQI*_3*D*_ trend with respect to the *MD*_3*D*_ behavior was obtained: for the metal sample, REVO X provided a higher *SQI*_3*D*_ (63.0, +33%) with respect to MIRACO NIR, whereas for the PLA sample, REVO X resulted in a lower *SQI*_3*D*_ (45.7, −22%) with respect to MIRACO NIR. Overall, these results indicate that while metrology-oriented systems can provide a substantial benefit on reflective surfaces when adequate surface preparation is performed, they may offer limited advantages on low-reflectivity surfaces.

As already discussed, the *SQI*_3*D*_ parameter was introduced to quantify the measurement accuracy of a scan in a reverse engineering perspective and to quantitatively support rapid scan acceptance/rejection decisions based on reproducible criteria. The formulation explicitly accounts for feature-dependent dimensional tolerances to reflect the different functional criticalities, and consequently the parameter can be tuned to operational requirements. At the same time, tolerance definition is a case- and operator-sensitive process and may influence the resulting *SQI*_3*D*_ values. For this reason, a Montecarlo sensitivity analysis was performed to quantify the impact of alternative tolerance sets on the parameter. A total of 200 tolerance scenarios were generated, each composed by *j* (number of geometrical features) integers independently sampled from a uniform distribution in the 1 ÷ 5 interval and assigned as tolerance of the corresponding dimensional feature. Two additional limit scenarios were included by setting all tolerances to 1% (most conservative) and 5% (most permissive). For each scenario, the *SQI*_3*D*_ was calculated for all the scans and its variability was analyzed through the resulting statistical distribution.

**Figure 14:**
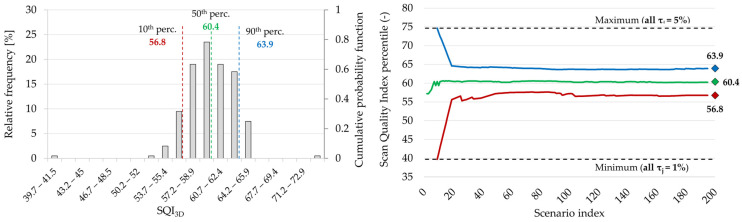
Montecarlo sensitivity analysis on Scan Quality Index (*SQI*_3*D*_) variation as a function of the tolerances set. (**Left**) *SQI*_3*D*_ statistical distribution obtained simulating 200 random tolerances (1 ÷ 5 interval) scenarios for the PLA, MIRACO NIR, SPRAY case. Vertical lines indicate the distribution 10th (red), 50th (green) and 90th (blue) percentiles (**Right**) stabilization plot for the 10th, 50th, 90th percentiles behavior over the simulated scenarios. Horizontal lines indicate the results for the two extreme scenario (all tolerances set to 1% or 5%).

Figure 14 shows an example of the statistical distribution (left) and the percentiles stabilization plot (right) for one representative case (PLA, MIRACO NIR, SPRAY), indicating that 200 scenarios are sufficient to characterize the statistical behavior of the parameter in the present dataset. Random tolerance scenarios resulted in a Gaussian *SQI*_3*D*_ distribution, while the two limit scenarios (all tolerances set to 1% or 5%) appeared as distant boundaries, as expected. Indeed, this behavior directly comes from the role of tolerances as a global scaling factor in the normalized scan score definition (*S_j_*, Equation (5)), based on operational needs. When all the geometrical features are assigned with the minimum tolerance (1%), the acceptable errors become uniformly small and the scan scores *S_j_* decrease simultaneously across the features, leading to very low *SQI*_3*D*_. Conversely, when all tolerances are set to 5% the acceptable errors increase for all the features and the scan scores *S_j_* tend to show higher values, leading to very high *SQI*_3*D*_. Therefore, these scenarios should be interpreted as worst- and best-case limits rather than representative operating conditions. Consistently with practical operational environments, the random scenarios mix tighter and larger tolerances across the features and, since the *SQI*_3*D*_ is calculated averaging many feature level scores, the aggregation naturally concentrates the results around an intermediate value, producing the observed Gaussian distribution.

**Figure 15:**
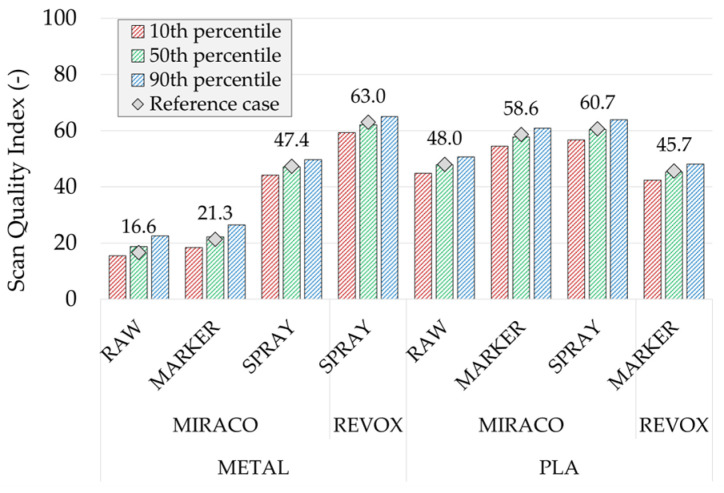
Montecarlo sensitivity analysis on Scan !uality index (*SQI*_3*D*_) variation as a function of the tolerances set: summary of the 10th, 50th, 90th percentiles of the *SQI*_3*D*_ distributions over the simulated scenarios for all the scan cases. The gray marker indicates the deterministic scenario.

Table 6 and Figure 15 report the *SQI*_3*D*_ results in terms of distribution percentiles (10th, 50th and 90th) obtained from the Monte Carlo tolerance scenarios for all cases. Within the investigated tolerance range (1–5%), the *SQI*_3*D*_ dispersion results limited across all the scan cases, with the percentile difference in the order of a few parameter units, typically ±3 around the median value corresponding to approximately ± 6% for *SQI*_3*D*_ ≈ 50. The magnitude of this variability is directly linked to the explored tolerance interval and would increase if wider or more heterogeneous tolerance ranges are considered. However, results show that the percentile-based statistics preserve the same scan comparative trends observed in the deterministic reference case. In particular, the scan-quality rank between surface conditions and between the two scanning systems remains unchanged across the distribution, indicating that *SQI*_3*D*_ keeps its significance using plausible tolerance variations and can therefore be used as a robust metric for system scan quality comparisons.

## 4. Discussion and Conclusions

The present study introduces a novel, quantitative indicator-driven framework to assess the real operational limits and potential of 3D scanning as an enabling technology for reverse engineering in shipyard environments, where material variability, surface conditions and the absence of laboratory grade workflows strongly influence achievable results.

The proposed scan-to-reference framework enables a holistic comparison of heterogeneous scans through the integration of three complementary indicators: (i) the Coverage Factor (*CF*_3*D*%_), quantifying scan geometric completeness; (ii) the Mean Distance (*MD*_3*D*_) between the scan and the CAD model, describing global geometrical agreement; (iii) the scan quality index (*SQI*_3*D*_) evaluating the dimensional fidelity in terms of measurement accuracy and variability. These parameters are defined to support rapid and reproducible scan acceptance/rejection decisions based on explicit criteria, rather than to provide a unique metrology evaluation. In this perspective, the framework is intended as a scanner-independent quality check to determine if an acquisition is sufficiently complete and dimensionally reliable to proceed with downstream reverse engineering tasks. Currently, the workflow is implemented on a set of parallel sections extracted consistently along a given direction, through a robust aggregation of completeness, deviation and measurement indicators at scan level. However, section extraction still requires non-negligible processing time and should be automated to support fast field deployment.

The method was applied to compare controlled acquisitions performed with two scanning systems: a field-oriented portable structured-light device (Revopoint MIRACO NIR) and a metrology-oriented fixed system (Revopoint REVOX). Acquisitions were performed on a purpose designed mechanical sample with geometrical features commonly encountered in shipyards components, including cavities, holes, grooves, sharp edges and curvature transitions. To evaluate the impact of different material reflectivity on reverse engineering processes, the sample was manufactured both in metal (reflective surface) using traditional CNC techniques and in PLA (low reflective surface) through additive manufacturing (FFF). Scans were performed on the as-is surfaces (i.e., raw) and with scan-tracking enhancement strategies (markers and scanning spray).

Overall, the three parameters provided a coherent and consistent assessment of scan quality. Raw reflective metal surfaces represented a critical condition for both systems: under this configuration, REVOX did not provide a usable reconstruction, while MIRACO NIR resulted in low *CF*_3*D*%_ (52.5 ± 12.2%) and *SQI*_3*D*_ (16.6) values together with a high *MD*_3*D*_ (0.96 ± 0.17 mm). In this context, surface preparation and scan-tracking enhancement methods strongly improved performance, with scanning spray providing the best benefit by increasing both *CF*_3*D*%_ (99.4 ± 0.4%, +89%) and *SQI*_3*D*_ (47.4, +186%), while reducing *MD*_3*D*_ (0.41 ± 0.02 mm, −57%). In addition, the reduction in the parameter variability along the sample thickness suggests a more homogeneous geometry reconstruction. These quantitative results are consistent with the qualitative inspection of the point clouds, where raw and marker-assisted conditions on metal exhibited discontinuities and the loss of fine features, whereas scanning spray enabled continuous and more homogeneous reconstruction.

For the low-reflectivity PLA sample, lower criticalities were observed for both systems, with generally better scan performance compared to the metal sample in all tested conditions. For instance, for raw surface condition *CF*_3*D*%_ increased to 96.6 ± 0.5% (+84%) and *SQI*_3*D*_ to 48.0 (+200%), while *MD*_3*D*_ decreased to 0.61 ± 0.01 mm (−36%). Consequently, under low-reflectivity conditions the benefit of tracking enhancement strategies remains, but is limited and mainly affects geometric uniformity and measurement consistency rather than coverage, which is already quite high in raw conditions; nevertheless, scanning spray provided the best overall performance.

The scanning systems comparison performed under comparable conditions (scanning spray for metal and marker assisted for PLA) showing different trends depending on the material. On the reflective metal sample, the metrology-oriented system (REVOX) provided clear benefits in geometrical reconstruction accuracy (*MD*_3*D*_: −32%, *SQI*_3*D*_: +33%) while achieving quite a complete scan coverage (*CF*_3*D*%_ = 99.3%). In contrast, the portable system (MIRACO NIR) achieved comparable or better performance on the less-reflective PLA sample. This outcome highlights that scanner performance cannot be interpreted independently of material properties and surface preparation strategy, and that in shipyard scenarios the workflow choices may affect the results as much as the scanning technology.

The results demonstrate that scan quality cannot be attributed to the scanning system alone, but emerges from a combination of material properties, optical behavior, sensor characteristics and procedural choices. From an operational standpoint, selecting the most suitable scanning solution involves a compromise between portability and ease of use on one side, and achievable metrological performance on the other, and should be driven by different variables such as material properties, surface finishing, component geometry and functional requirements of the reconstructed model. Portable, field-oriented scanners can provide acceptable results in terms of completeness and geometrical accuracy when surfaces are optically favorable or when an effective tracking strategy is adopted, offering advantages in setup time and usability that are critical in operative situations. Conversely, when scanning reflective components and when dimensional accuracy is critical, metrology-oriented systems combined with appropriate surface preparation represent the most reliable option.

From a methodological point of view, since all the proposed quality indicators are computed from scan to CAD section comparisons, the proposed framework relies on the availability of an accurate reference model and a stable alignment between scan and reference. Any potential discrepancy between the reference geometry and the true component may propagate into all the indicators, introducing uncontrolled artifacts. In addition, any potential misalignment bias may distort deviation and coverage estimates, especially when the geometry contains locally ambiguous features. Moreover, the workflow remains sensitive to surface reflectivity, texture, accessibility and scanning geometry, which can induce local tracking losses, non-uniform point density and section-to-section variability. Finally, while *SQI*_3*D*_ is intentionally formulated through feature-specific tolerances, defining scan acceptance thresholds is an application-dependent process and may require an initial calibration phase, like iterative tuning against functional needs and manufacturing constraints. Nevertheless, within realistic tolerance ranges the sensitivity analysis showed that *SQI*_3*D*_ remains sufficiently stable to preserve the comparative ranking between scanning configurations. From a practical point of view, the current workflow implementation works on parallel sections extracted from the reconstructed points cloud. Preprocessing and section extraction still requires non-negligible operator time and should be automated to support fast field deployment.

As discussed above, the workflow is currently suited for components with at least one homogenous dimension, for which a set of consistent parallel sections can be extracted and aggregated. For more complex geometries, the same indicators remain applicable, but the sampling strategy becomes more critical, as additional sections or feature driven sampling regions may be required to achieve representative coverage of functionally relevant areas. When consistent section aggregation is not feasible (i.e., limited homogeneous regions or strongly varying cross-sections), indicators can still be computed on individual sections; however, the reduced redundancy limits the possibility of averaging and increases sensitivity to local alignment and measurement variability, thereby increasing the uncertainty of the overall quality assessment.

This work, carried out within a research project aimed at introducing specific operative innovations to support the digital transition of shipbuilding operations, delivers a practical and transferable method for assessing the dimensional reliability of reverse engineering-oriented 3D scanning under shipyard-relevant constraints, such as limited accessibility, time pressure and operator variability. Importantly, the framework can act as a standardized quality check step, enabling objective comparison of heterogeneous acquisitions and supporting rapid acceptance or rejection decisions based on reproducible criteria. Future works will focus on two complementary directions: (i) a detailed metrological characterization of the investigated scanning systems, including uncertainty budget analysis as a function of the scanning conditions and (ii) extension of the proposed methodology to a wider range of geometries, with focus on the needed modification for applications on more complex samples, and operating scenarios, as the basis for the development of potential automated on-line tools that link scan quality to downstream requirements and support RE decisions through explicit acceptance criteria based on objective and reproducible scan indicators.

## Figures and Tables

**Table 1:** Details of the geometrical features’ dimensional characterization performed by a caliper (0.05 mm resolution) for both PLA and metal mechanical samples.

Feature	Dimension /mm		Feature	Dimension /mm		Feature	Dimension /mm	
	PLA	Metal		PLA	Metal		PLA	Metal
L_(x)_	165.00	167.10	G1_D_(x)_	32.50	32.60	G4_L_(x)_	10.00	10.00
W_(y)_	100.00	98.15	G1_L_(x)_	10.05	10.05	G4_D_(y)_	4.05	4.10
H1_C_(x)_	127.05	128.90	G1_D_(y)_	1.00	1.00	G45_(x)_	6.00	6.05
H1_C_(y)_	50.00	49.00	G12_(x)_	3.00	2.95	G5_L_(x)_	10.00	10.10
H1_Φ	36.00	36.00	G2_L_(x)_	10.05	10.10	G5_D_(y)_	5.05	5.20
H1_G_(x)_	6.00	6.25	G2_D_(y)_	2.05	2.05	G56_(x)_	7.00	7.05
H1_G_(y)_	10.05	8.00	G23_(x)_	4.00	4.00	G6_L_(x)_	10.00	10.00
H2_C_(x)_	57.05	58.65	G3_L_(x)_	10.05	10.10	G6_D_(y)_	6.00	6.15
H2_C_(y)_	70.00	67.90	G3_D_(y)_	3.00	3.10	S_(z)_	15.05	15.00
H2_Φ	20.00	20.00	G34_(x)_	5.00	5.10	H_(z)_	65.00	65.15

**Table 2:** Details of the main specifications of the Revopoint 3D scanner used for experimental activity: MIRACO NIR (portable structured-light scanner) and METRO X (metrology-oriented system integrating multiple scanning technologies).

Feature	MIRACO NIR	METRO X
Scanning Technology	IR structured light with quad-depth cameras	Hybrid: 14× laser lines, 62× structured-light lines
Precision	Up to 0.02 mm	Up to 0.01 mm
Accuracy	Up to 0.05 mm	Up to 0.03 mm (3D: 0.03 mm + 0.1 × L)
Field of View	28 × 53 mm to 975 × 775 mm	160 × 70 mm to 320 × 215 mm
Scanning Modes	Single and continuous	Manual, tripod, dual-axis turntable
Operation	Standalone with built-in processing	Requires external PC with GPU
Display	6″ AMOLED touchscreen (2K)	None (uses external monitor)
Portability	High (750 g, built-in battery, Wi-Fi 6)	Medium (508 g, USB-C powered, requires PC)

**Table 3:** Summary of the quantitative parameters obtained for both scanning systems (MIRACO NIR and REVO X) on metal and PLA samples with different surface conditions.

Sample	Scan System	Surface Condition	Coverage Factor (*CF*_3*D*%_)			Cloud to CAD Distance (*MD*_3*D*_)			Scan Quality Index (*SQI*_3*D*_)	
			Mean (%)	St. Dev. (%)	Increment (%)	Mean /mm	St. Dev. /mm	Increment (%)	Mean -	Increment (%)
Metal	MIRACO NIR	Raw	52.5	12.2	-	0.96	0.17	-	16.6	-
		Marker	59.4	16.9	13.1	0.51	0.07	46.9	21.3	29
		Spray	99.4	0.4	89.2	0.41	0.02	57.0	47.4	186
	REVO X	Spray	99.3	5.7	−0.1	0.28	0.04	32.3	63.0	33
PLA	MIRACO NIR	Raw	96.6	0.5	-	0.61	0.01	-	48.0	-
		Marker	93.2	5.0	−3.5	0.36	0.03	40.8	58.6	22
		Spray	99.3	0.7	2.8	0.26	0.02	57.4	60.7	27
	REVO X	Marker	99.2	0.7	6.5	0.53	0.05	−46.3	45.7	−22

**Table 4:** Dimensional characterization from the scanned sections obtained for the metal sample with both scanning systems and different surface conditions: estimated measures were defined as the mean value over the seven sections, with standard deviation showing the variability over the sample thickness.

Feature		Nominal Value /mm	MIRACO NIR						REVO X	
			Raw		Marker		Spray		Spray	
			Mean	St. Dev.	Mean	St. Dev.	Mean	St. Dev.	Mean	St. Dev.
General	L_(x)_	167.1	164.8	1.22	166.3	0.29	166.3	0.18	165.8	0.37
	W_(y)_	98.1	97.0	4.35	94.6	17.00	97.5	0.07	98.0	0.17
Holes	H1_C_(x)_	128.9	128.5	0.38	128.3	0.50	128.6	0.17	128.2	0.08
	H1_C_(y)_	49.0	49.0	0.32	50.2	1.40	49.1	0.10	48.9	0.03
	H1_Φ	36.0	37.0	0.56	35.4	0.81	36.5	0.25	35.7	0.04
	H1_G_(y)_	8.0	9.1	0.60	0.0	0.00	9.2	1.62	6.7	2.94
	H2_C_(x)_	58.6	58.6	0.38	57.9	0.20	58.5	0.30	58.0	0.09
	H2_C_(y)_	67.9	67.9	0.21	67.9	0.12	67.9	0.10	67.9	0.11
	H2_Φ	20.0	20.9	0.69	19.9	0.33	20.2	0.48	19.6	0.13
Grooves	G1_D_(x)_	32.6	-	-	-	-	32.1	0.28	30.1	0.25
	G1_L_(x)_	10.1	-	-	-	-	10.2	0.03	10.0	0.05
	G1_D_(y)_	1.0	-	-	-	-	1.0	0.04	1.0	0.03
	G12_(x)_	3.0	-	-	-	-	3.2	0.11	2.9	0.03
	G2_L_(x)_	10.1	-	-	-	-	9.8	0.23	10.2	0.21
	G2_D_(y)_	2.1	-	-	-	-	1.9	0.08	1.8	0.07
	G23_(x)_	4.0	-	-	-	-	4.3	0.19	5.0	0.21
	G3_L_(x)_	10.1	-	-	-	-	9.5	0.36	10.0	0.08
	G3_D_(y)_	3.1	-	-	-	-	3.0	0.16	3.1	0.04
	G34_(x)_	5.1	-	-	-	-	5.7	0.37	5.1	0.07
	G4_L_(x)_	10.0	-	-	-	-	9.6	0.63	9.9	0.08
	G4_D_(y)_	4.1	-	-	-	-	4.0	0.08	4.1	0.04
	G45_(x)_	6.0	-	-	-	-	6.3	0.62	6.0	0.09
	G5_L_(x)_	10.1	-	-	-	-	9.3	0.68	9.9	0.09
	G5_D_(y)_	5.2	-	-	4.2	1.07	5.0	0.05	5.1	0.08
	G56_(x)_	7.0	-	-	6.9	0.74	7.6	0.67	7.1	0.08
	G6_L_(x)_	10.0	-	-	9.7	0.98	9.5	0.63	10.0	0.06
	G6_D_(y)_	6.1	-	-	6.1	0.28	6.0	0.06	6.1	0.04

**Table 5:** Dimensional characterization from the scanned sections obtained for PLA sample with both scanning systems and different surface conditions: estimated measures were defined as the mean value over the seven sections, with standard deviation showing the variability over the sample thickness.

Feature		Nominal Value /mm	MIRACO NIR						REVO X	
			Raw		Marker		Spray		Marker	
			Mean	St. Dev.	Mean	St. Dev.	Mean	St. Dev.	Mean	St. Dev.
General	L_(x)_	165.0	166.4	0.34	164.6	1.05	166.3	0.15	164.0	0.38
	W_(y)_	100.0	99.1	0.30	99.7	0.24	100.8	0.04	99.3	0.20
Holes	H1_C_(x)_	127.0	127.4	0.03	126.8	0.06	127.3	0.03	126.7	0.10
	H1_C_(y)_	50.0	49.9	0.01	49.7	0.08	50.1	0.06	50.1	0.19
	H1_Φ	36.0	36.7	0.03	36.8	0.12	35.6	0.04	36.5	0.12
	H1_G_(y)_	10.0	10.8	0.31	11.1	0.50	9.6	0.24	10.2	0.17
	H2_C_(x)_	57.0	57.6	0.18	56.8	0.11	57.4	0.04	56.8	0.06
	H2_C_(y)_	70.0	69.8	0.14	69.8	0.09	70.1	0.04	69.9	0.11
	H2_Φ	20.0	20.4	0.06	19.7	0.18	19.5	0.05	20.6	0.16
Grooves	G1_D_(x)_	32.5	32.5	0.43	32.1	0.24	33.7	0.03	31.5	0.28
	G1_L_(x)_	10.0	10.1	0.03	10.0	0.05	9.4	0.30	10.0	0.33
	G1_D_(y)_	1.0	1.0	0.03	1.0	0.13	1.1	0.04	0.9	0.18
	G12_(x)_	3.0	2.7	0.04	3.2	0.05	3.4	0.31	3.2	0.42
	G2_L_(x)_	10.0	10.1	0.05	9.8	0.05	9.1	0.09	10.0	0.45
	G2_D_(y)_	2.0	2.1	0.08	1.8	0.03	2.0	0.03	1.8	0.35
	G23_(x)_	4.0	4.7	0.46	4.3	0.07	4.8	0.15	3.9	0.23
	G3_L_(x)_	10.0	9.2	0.05	9.8	0.08	9.3	0.12	10.3	0.25
	G3_D_(y)_	3.0	3.2	0.12	2.9	0.03	3.0	0.02	3.0	0.17
	G34_(x)_	5.0	5.8	0.06	4.9	0.09	5.8	0.14	4.6	0.20
	G4_L_(x)_	10.0	9.2	0.18	9.7	0.26	9.2	0.14	10.2	0.20
	G4_D_(y)_	4.0	4.1	0.03	4.0	0.05	4.0	0.06	3.7	0.29
	G45_(x)_	6.0	6.8	0.09	6.2	0.27	6.8	0.07	5.5	0.21
	G5_L_(x)_	10.0	9.2	0.08	9.9	0.14	9.3	0.09	10.5	0.17
	G5_D_(y)_	5.0	4.9	0.06	5.0	0.03	5.0	0.11	4.9	0.36
	G56_(x)_	7.0	7.9	0.05	7.1	0.12	7.8	0.18	6.3	0.15
	G6_L_(x)_	10.0	9.1	0.05	9.9	0.19	9.3	0.13	10.7	0.16
	G6_D_(y)_	6.0	5.9	0.05	6.0	0.07	5.9	0.02	5.8	0.15

**Table 6:** Montecarlo sensitivity analysis on Scan Quality index (*SQI*_3*D*_) variation as a function of the tolerances set: summary of the *SQI*_3*D*_ statistical distribution parameters over the simulated scenarios for all the scan cases.

Sample	Scan System	Surface Condition	Scan Quality Index (*SQI*_3*D*_)						
			Mean	St. Dev.	Minimum	10th Percentile	50th Percentile	90th Percentile	Maximum
Metal	MIRACO NIR	Raw	18.8	1.3	14.1	15.4	18.8	22.5	25.7
		Marker	22.3	1.5	16.1	18.4	22.2	26.5	30.8
		Spray	46.9	2.5	30.5	44.2	47.0	49.7	58.5
	REVO X	Spray	62.1	2.7	43.9	59.3	62.1	65.0	73.4
PLA	MIRACO NIR	Raw	47.8	2.6	30.7	44.8	47.8	50.7	59.6
		Marker	57.7	2.8	39.1	54.4	57.7	60.8	69.8
		Spray	60.3	3.2	39.7	56.8	60.4	63.9	74.7
	REVO X	Marker	45.3	2.6	28.9	42.4	45.2	48.1	57.2

## Data Availability

The original contributions presented in this study are included in the article. Further inquiries can be directed to the corresponding author.

## Abbreviations

The following abbreviations are used in this manuscript:

RE	Reverse Engineering
AM	Additive Manufacturing
CAD	Computer-Aided Design
CNC	Computerized Numerical Control
FFF	Fused Filament Fabrication
PLA	PolyLactic Acid
ICP	Iterative Closest Point
